# Supplementary material for: Quantifying BRCA1 and BRCA2 mRNA Isoform Expression Levels in Single Cells
Source: Int J Mol Sci. 2019 Feb 6;20(3):693. doi: 10.3390/ijms20030693 (PMC6387195; doi:10.3390/ijms20030693)
Supplement: Supplementary file 1 [file ijms-20-00693-s001.pdf]

Supplementary information

RNAscope probe design

Z probe pairs were designed to target specific *BRCA1* and *BRCA2* alternative isoforms, so that RNAscope could be used to detect and compare the associated expression levels between variant carriers and controls. These site-specific oligonucleotides are each detectable in separate colour channels, referred to as C1 (conjugated to Alexa 488 - Green) and C2 (conjugated to Alexa 550 - Red).

Selection of targeted isoforms was restricted by the requirements of the probes. Ten to 20 probes are needed for optimal signalling, which in turn necessitates a stretch of mRNA between 500 and 1,000 nt long within the targeted skipping event. This meant that events less than 500 nt long are not adequate for optimal detection with this technology. *BRCA1* C1 probes were designed to target exon 11 (3426 nt), and C2 probes to target exons 1-10 (670nt) (from here on these *BRCA1*-specific probes are referred to as C11 and C1-10, respectively). *BRCA2* C1 probes were designed to target exons 17/18 (526 nt), and C2 to target exon 11-16 (5,896nt) (from here on *BRCA2*-specific probes are referred to as C17-18 and C11-16, respectively) (Supplementary Figure 1; Panels 1a and 2a). Probes were designed so that *BRCA1* C11 probe binding will not be observed for transcripts with exon 11 skipping, while *BRCA2* C17-18 probe binding would not be observed for transcripts with exons 17-18 skipping. Transcripts with detectable binding for both probes do not carry the targeted deletion. While the probe locations were specified by the candidate to fit the requirements of this project, RNAscope probes are created within the founding organisation and the precise sequences are proprietary (Advanced Cell Diagnostics, Newark, CA).

Cell adhesion and hybridisation preparation

Briefly, cultured LCL samples were harvested by centrifugation (400 g), washed with PBS, resuspended in formalin (10%) and incubated at 37°C for one hour to fix the cells for analysis. Cells were then centrifuged (1000 rpm), washed in PBMC-Wash (AcdBio) and resuspended in 70% ethanol ready for fixing to slides. Suspended cells were transferred into pre-assembled cyto-centrifuge cartridges (Thermo Fisher Scientific), spun in a Shandon CytoSpin II

Cytocentrifuge (Shandon Scientific, Cheshire, UK) at 800 RCF for 20 minutes, and then air dried.

Slides were prepared for pre-treatment with four consecutive 5 minute ethanol incubation steps at room temperature (50% ethanol twice, followed by 70% and 100%). Slides were subsequently dried at 37°C for 30 minutes in a HybEZ™ oven (AcdBio). All following incubations were completed in the HybEZ™ oven in a humidity control tray to prevent the samples drying out. Cell spots were circled with a hydrophobic barrier pen to contain treatments and hybridisation mix across the cell spread. Pretreat 3 solution was then added, covering each cell spot entirely, before incubation at 40°C for 30 minutes. Slides were washed in PBS prior to probe hybridisation.

Probe hybridisation and slide mounting

Gene-specific C1 and C2 probes were incubated at 40°C in the HybEZ™ oven for 10 minutes, then left to cool to room temperature before mixing at a 1:50 ratio. The prepared probes, and those provided for the positive and negative controls, were pipetted onto their respective samples to completely cover the cells, prior to hybridisation in a two hour 40°C incubation. Four 40°C incubation steps followed to consecutively hybridise several amplifiers to these probes. Each sample slide (including controls) was first incubated with AMP 1-FL for 30 minutes, followed by AMP 2-FL for 15 minutes, AMP 3-FL for 30 minutes and AMP 4-FL for 15 minutes. Each of these hybridisation steps alternated with a washing step involving two sequential 2 minute incubations at room temperature in fresh wash buffer. Cells were then counterstained with DAPI via a 30 second incubation at room temperature. Anti-fading solution (2 mg/mL p-phenylenediamine (Sigma-Aldrich, New Zealand) in 80% glycerol pH 7.8 (ice cold solution)) to prevent photo-bleaching was then applied as the mounting medium before a cover slip was immediately placed over the cells on each slide. Positive and negative controls are shown in Supplementary Figures 2 and 3.

Supplementary Table 1. *BRCA1* mRNA isoforms detected in *BRCA1* c.671-2 A>G.

	***BRCA1* c.671-2 A>G Treated**					***BRCA1* c.671-2 A>G Untreated**				
	**Red TRITC**	**Green FITC**	**FL**	**Delta 11**	**Total**	**Red TRITC**	**Green FITC**	**FL**	**Delta 11**	**Total**
1	6	6	6	0	6	7	7	7	0	7
2	6	5	5	1	6	4	6	6	0	6
3	5	5	5	0	5	0	2	2	0	2
4	3	7	7	0	7	0	5	5	0	5
5	3	8	8	0	8	0	0	0	0	0
6	0	1	1	0	1	0	0	0	0	0
7	1	1	1	0	1	0	0	0	0	0
8	1	4	4	0	4	0	0	0	0	0
9	6	1	1	5	6	0	0	0	0	0
10	3	3	3	0	3	0	0	0	0	0
11	1	5	5	0	5	0	0	0	0	0
12	4	0	0	4	4	0	0	0	0	0
13	3	0	0	3	3	0	0	0	0	0
14	1	0	0	1	1	0	0	0	0	0
15	1	0	0	1	1	0	0	0	0	0
16	17	9	9	8	17	0	0	0	0	0
17	12	9	9	3	12	0	0	0	0	0
18	4	6	6	0	6	0	0	0	0	0
19	1	1	1	0	1	0	0	0	0	0
20	4	4	4	0	4	0	0	0	0	0
21	2	2	2	0	2	0	0	0	0	0
22	1	1	1	0	1	0	0	0	0	0
23	0	0	0	0	0	0	0	0	0	0
24	0	0	0	0	0	0	0	0	0	0
25	0	0	0	0	0	0	0	0	0	0
26	0	0	0	0	0	0	0	0	0	0
27	0	0	0	0	0	0	0	0	0	0
28	0	0	0	0	0	0	0	0	0	0
29	0	0	0	0	0	0	0	0	0	0
30	0	0	0	0	0	0	0	0	0	0
31	0	0	0	0	0	0	0	0	0	0
32	0	0	0	0	0	0	0	0	0	0
33	0	0	0	0	0	0	0	0	0	0
34	0	0	0	0	0	0	0	0	0	0
35	0	0	0	0	0	0	0	0	0	0
36	0	0	0	0	0	0	0	0	0	0
37	0	0	0	0	0	0	0	0	0	0
38	0	0	0	0	0	0	0	0	0	0
39	0	0	0	0	0	0	0	0	0	0
40	0	0	0	0	0	0	0	0	0	0
41	0	0	0	0	0	0	0	0	0	0
42	0	0	0	0	0	0	0	0	0	0
43	0	0	0	0	0	0	0	0	0	0
44	0	0	0	0	0	0	0	0	0	0
45	0	0	0	0	0	0	0	0	0	0
46	0	0	0	0	0	0	0	0	0	0
47	0	0	0	0	0	0	0	0	0	0
48	0	0	0	0	0	0	0	0	0	0
49	0	0	0	0	0	0	0	0	0	0
50	0	0	0	0	0	0	0	0	0	0
**Total (% Delta)**	**85**	**78**	**78**	**26**	**104(25%)**	**11**	**20**	**20**	**0**	**20(0%)**

Supplementary Table 2. *BRCA1* mRNA isoforms detected in the control.

	Control Treated					Control Untreated				
	Red TRITC	Green FITC	FL	Delta 11	Total	Red TRITC	Green FITC	FL	Delta 11	Total
1	10	5	5	5	10	0	2	2	0	2
2	0	3	3	0	3	0	4	4	0	4
3	0	0	0	0	0	0	2	2	0	2
4	0	0	0	0	0	1	1	1	0	1
5	0	0	0	0	0	2	2	2	0	2
6	0	0	0	0	0	0	0	0	0	0
7	0	0	0	0	0	0	0	0	0	0
8	0	0	0	0	0	0	0	0	0	0
9	0	0	0	0	0	0	0	0	0	0
10	0	0	0	0	0	0	0	0	0	0
11	0	0	0	0	0	0	0	0	0	0
12	0	0	0	0	0	0	0	0	0	0
13	0	0	0	0	0	0	0	0	0	0
14	0	0	0	0	0	0	0	0	0	0
15	0	0	0	0	0	0	0	0	0	0
16	0	0	0	0	0	0	0	0	0	0
17	0	0	0	0	0	0	0	0	0	0
18	0	0	0	0	0	0	0	0	0	0
19	0	0	0	0	0	0	0	0	0	0
20	0	0	0	0	0	0	0	0	0	0
21	0	0	0	0	0	0	0	0	0	0
22	0	0	0	0	0	0	0	0	0	0
23	0	0	0	0	0	0	0	0	0	0
24	0	0	0	0	0	0	0	0	0	0
25	0	0	0	0	0	0	0	0	0	0
26	0	0	0	0	0	0	0	0	0	0
27	0	0	0	0	0	0	0	0	0	0
28	0	0	0	0	0	0	0	0	0	0
29	0	0	0	0	0	0	0	0	0	0
30	0	0	0	0	0	0	0	0	0	0
31	0	0	0	0	0	0	0	0	0	0
32	0	0	0	0	0	0	0	0	0	0
33	0	0	0	0	0	0	0	0	0	0
34	0	0	0	0	0	0	0	0	0	0
35	0	0	0	0	0	0	0	0	0	0
36	0	0	0	0	0	0	0	0	0	0
37	0	0	0	0	0	0	0	0	0	0
38	0	0	0	0	0	0	0	0	0	0
39	0	0	0	0	0	0	0	0	0	0
40	0	0	0	0	0	0	0	0	0	0
41	0	0	0	0	0	0	0	0	0	0
42	0	0	0	0	0	0	0	0	0	0
43	0	0	0	0	0	0	0	0	0	0
44	0	0	0	0	0	0	0	0	0	0
45	0	0	0	0	0	0	0	0	0	0
46	0	0	0	0	0	0	0	0	0	0
47	0	0	0	0	0	0	0	0	0	0
48	0	0	0	0	0	0	0	0	0	0
49	0	0	0	0	0	0	0	0	0	0
50	0	0	0	0	0	0	0	0	0	0
**Totals (% Delta)**	**10**	**8**	**8**	**5**	**13(38%)**	**3**	**11**	**11**	**0**	**11(0%)**

Supplementary Table 3. *BRCA2* mRNA isoforms detected in the *BRCA2* c.7988 A>T.

	BRCA2 c.7988 A>T Treated					*BRCA2* c.7988 A>T Untreated				
	Red TRITC	Green FITC	FL	Delta 17,18	Total (% Delta)	Red TRITC	Green FITC	FL	Delta 17,18	Total (% Delta)
1	1	1	1	0	1	7	2	2	5	7
2	3	1	1	2	3	3	1	1	2	3
3	1	1	1	0	1	8	6	6	2	8
4	3	0	0	3	3	1	1	1	0	1
5	8	5	5	3	8	4	0	0	4	4
6	9	4	4	5	9	5	5	5	0	5
7	0	1	1	0	1	7	2	2	5	7
8	1	1	1	0	1	3	0	0	3	3
9	4	1	1	3	4	6	6	6	0	6
10	4	4	4	0	4	2	0	0	2	2
11	1	2	2	0	2	3	0	0	3	3
12	1	2	2	0	2	8	1	1	7	8
13	5	6	6	0	6	4	3	3	1	4
14	0	1	1	0	1	1	1	1	0	1
15	0	4	4	0	4	1	0	0	1	1
16	0	1	1	0	1	2	0	0	2	2
17	14	14	14	0	14	2	1	1	1	2
18	0	12	12	0	12	6	1	1	5	6
19	3	2	2	1	3	6	2	2	4	6
20	1	1	1	0	1	4	2	2	2	4
21	24	11	11	13	24	1	0	0	1	1
22	7	3	3	4	7	3	1	1	2	3
23	7	1	1	6	7	4	2	2	2	4
24	7	1	1	6	7	5	6	6	0	6
25	29	8	8	21	29	0	0	0	0	0
26	1	1	1	0	1	0	0	0	0	0
27	1	0	0	1	1	0	0	0	0	0
28	2	0	0	2	2	0	0	0	0	0
29	1	0	0	1	1	0	0	0	0	0
30	0	0	0	0	0	0	0	0	0	0
31	0	0	0	0	0	0	0	0	0	0
32	0	0	0	0	0	0	0	0	0	0
33	0	0	0	0	0	0	0	0	0	0
34	0	0	0	0	0	0	0	0	0	0
35	0	0	0	0	0	0	0	0	0	0
36	0	0	0	0	0	0	0	0	0	0
37	0	0	0	0	0	0	0	0	0	0
38	0	0	0	0	0	0	0	0	0	0
39	0	0	0	0	0	0	0	0	0	0
40	0	0	0	0	0	0	0	0	0	0
41	0	0	0	0	0	0	0	0	0	0
42	0	0	0	0	0	0	0	0	0	0
43	0	0	0	0	0	0	0	0	0	0
44	0	0	0	0	0	0	0	0	0	0
45	0	0	0	0	0	0	0	0	0	0
46	0	0	0	0	0	0	0	0	0	0
47	0	0	0	0	0	0	0	0	0	0
48	0	0	0	0	0	0	0	0	0	0
49	0	0	0	0	0	0	0	0	0	0
50	0	0	0	0	0	0	0	0	0	0
**Total**	**138**	**89**	**89**	**71**	**160(44%)**	**96**	**43**	**43**	**54**	**97(56%)**

Supplementary Table 4. *BRCA2* mRNA isoforms detected in a control using RNAscope.

	Control Treated						Control Untreated					
	Red TRITC	Green FITC		FL	Delta 17,18	Total	Red TRITC	Green FITC		FL	Delta 17,18	Total
1	2	0		0	2	2	1	1		1	0	1
2	5	5		5	0	5	1	1		1	0	1
3	1	1		1	0	1	1	1		1	0	1
4	1	0		0	1	1	1	0		0	1	1
5	2	0		0	2	2	3	2		2	1	3
6	4	4		4	0	4	0	0		0	0	0
7	2	1		1	1	2	0	0		0	0	0
8	3	2		2	1	3	0	0		0	0	0
9	5	5		5	0	5	0	0		0	0	0
10	4	4		4	0	4	0	0		0	0	0
11	0	1		1	0	1	0	0		0	0	0
12	1	1		1	0	1	0	0		0	0	0
13	0	0		0	0	0	0	0		0	0	0
14	0	0		0	0	0	0	0		0	0	0
15	0	0		0	0	0	0	0		0	0	0
16	0	0		0	0	0	0	0		0	0	0
17	0	0		0	0	0	0	0		0	0	0
18	0	0		0	0	0	0	0		0	0	0
19	0	0		0	0	0	0	0		0	0	0
20	0	0		0	0	0	0	0		0	0	0
21	0	0		0	0	0	0	0		0	0	0
22	0	0		0	0	0	0	0		0	0	0
23	0	0		0	0	0	0	0		0	0	0
24	0	0		0	0	0	0	0		0	0	0
25	0	0		0	0	0	0	0		0	0	0
26	0	0		0	0	0	0	0		0	0	0
27	0	0		0	0	0	0	0		0	0	0
28	0	0		0	0	0	0	0		0	0	0
29	0	0		0	0	0	0	0		0	0	0
30	0	0		0	0	0	0	0		0	0	0
31	0	0		0	0	0	0	0		0	0	0
32	0	0		0	0	0	0	0		0	0	0
33	0	0		0	0	0	0	0		0	0	0
34	0	0		0	0	0	0	0		0	0	0
35	0	0		0	0	0	0	0		0	0	0
36	0	0		0	0	0	0	0		0	0	0
37	0	0		0	0	0	0	0		0	0	0
38	0	0		0	0	0	0	0		0	0	0
39	0	0		0	0	0	0	0		0	0	0
40	0	0		0	0	0	0	0		0	0	0
41	0	0		0	0	0	0	0		0	0	0
42	0	0		0	0	0	0	0		0	0	0
43	0	0		0	0	0	0	0		0	0	0
44	0	0		0	0	0	0	0		0	0	0
45	0	0		0	0	0	0	0		0	0	0
46	0	0		0	0	0	0	0		0	0	0
47	0	0		0	0	0	0	0		0	0	0
48	0	0		0	0	0	0	0		0	0	0
49	0	0		0	0	0	0	0		0	0	0
50	0	0		0	0	0	0	0		0	0	0
**Total (% Delta)**	**30**	**24**		**24**	**7**	**31 (22%)**	**7**	**5**		**5**	**2**	**7(29%)**

Supplementary Table 5. Fluorescently labelled mRNA counts compared between cells containing splice disrupting variants and controls. Samples are not treated with NMD inhibitor cycloheximide. The P-value for *BRCA1* c.671-2 A>G was not able to be calculated due to zero values from low signal detection.

**NMD untreated**	Total gene-specific mRNA isoforms detected[a]	Number of targeted alternative transcripts detected[b]		
Control	11	0	**p-value**[c] **=**	Undefined
BRCA1 c.671-2 A>G	20	0	**OR**[d]**=**	0.000
Control	5	2	**p-value**[c] **=**	0.319
BRCA2 c.7988 A>T	43	54	**OR**[d]**=**	3.140

[a] Number of signals from *BRCA1* exon and exon 1-10 probes, or exon 11 probes only, for *BRCA1*-targeting probes, or the number of signals from *BRCA2* exon 17-18 and exon 11-16 probes, or exon 17-18 probes only, for the *BRCA2*-targeting probes. [b] Number of signals from *BRCA1* exon 1-10 probes only (Δ11 transcripts), or the number of signals from *BRCA2* exon 11-16 probes only (Δ17-18 transcripts). [c] Yates corrected chi-squared test. [d] OR: Odds ratio.

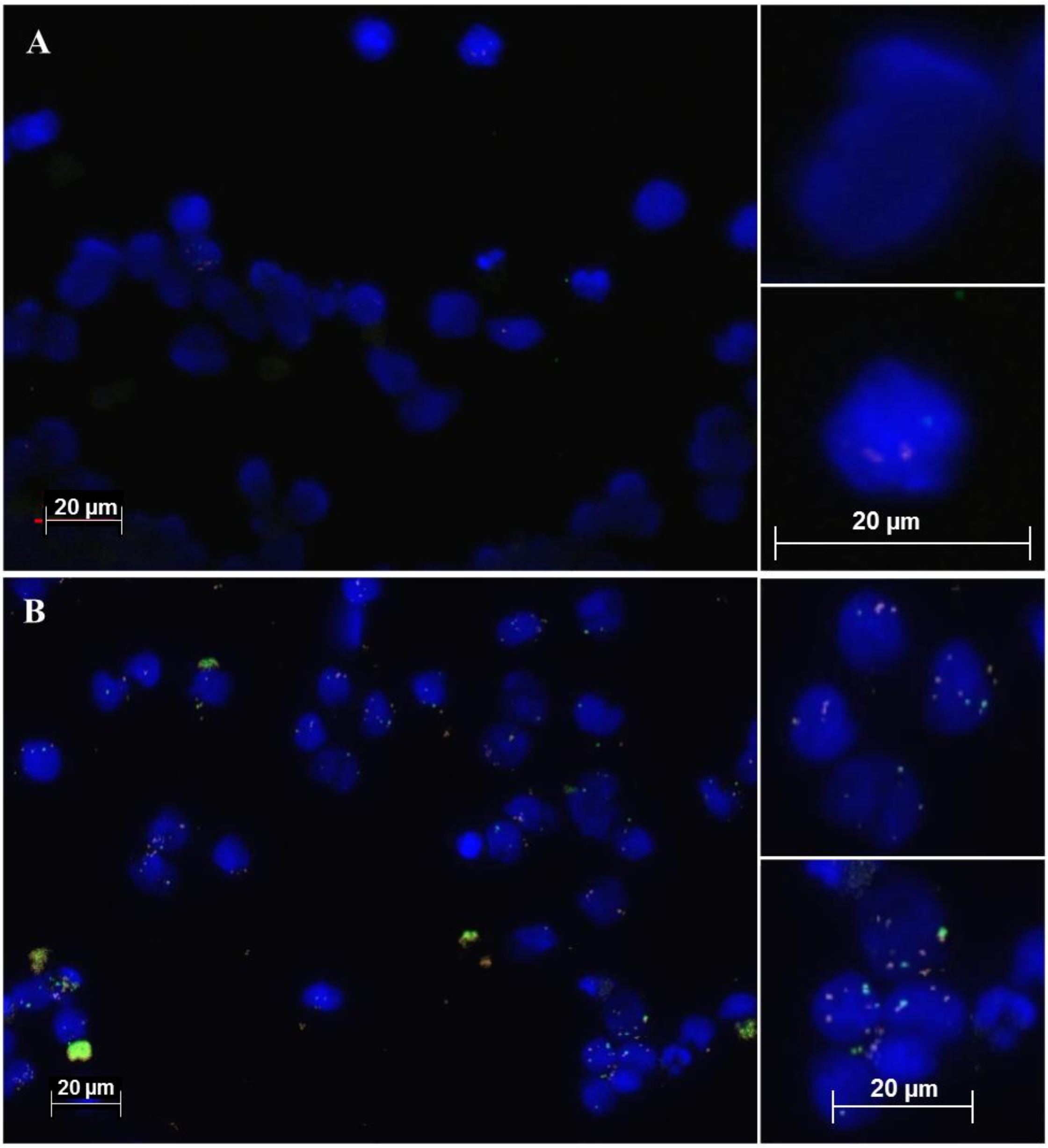

Supplementary Figure 1. Panel A: *BRCA1* negative control. Panel B: *BRCA1* positive control.

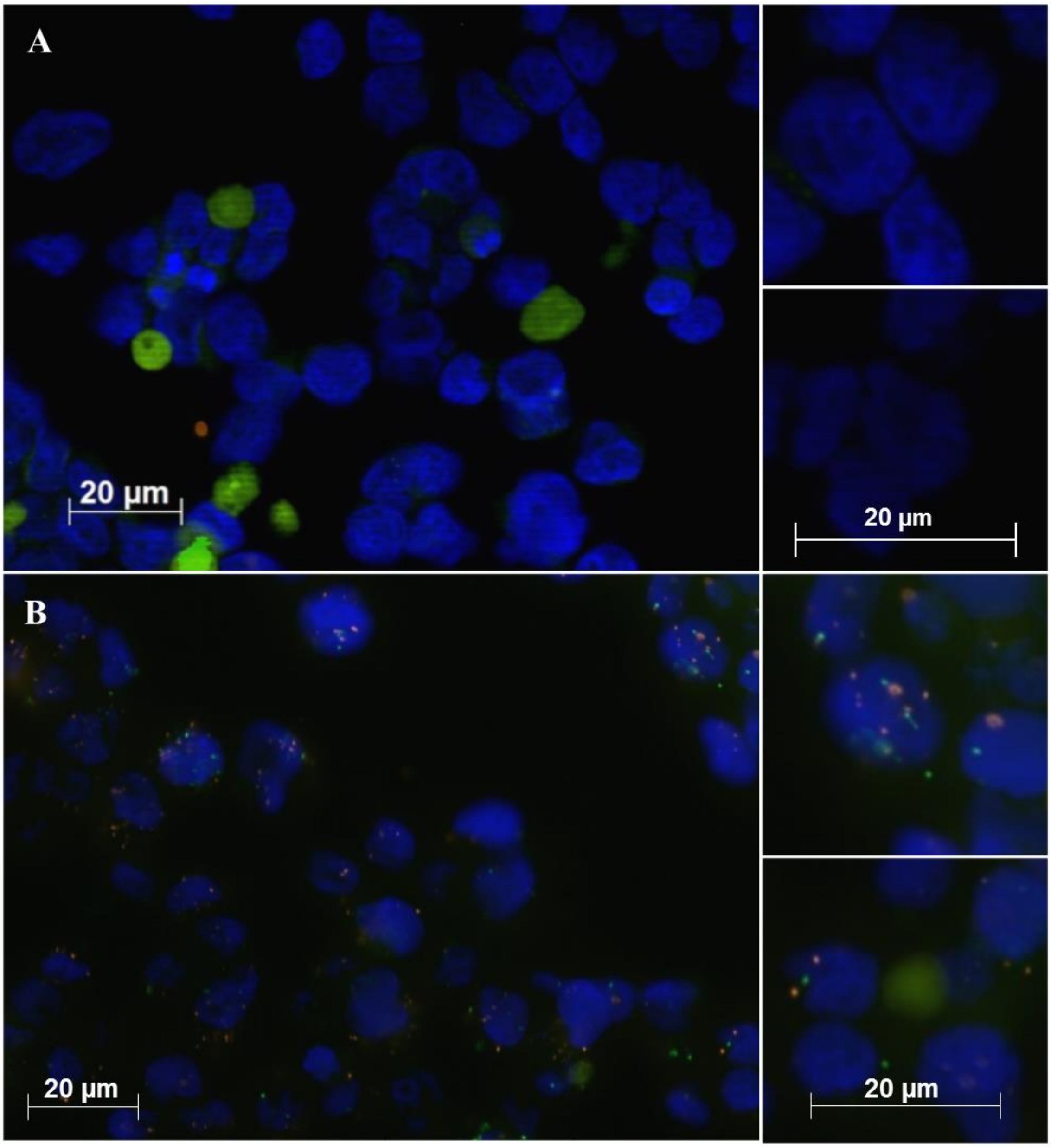

Supplementary Figure 2. Panel A: *BRCA2* negative control. Panel B: *BRCA2* positive control.

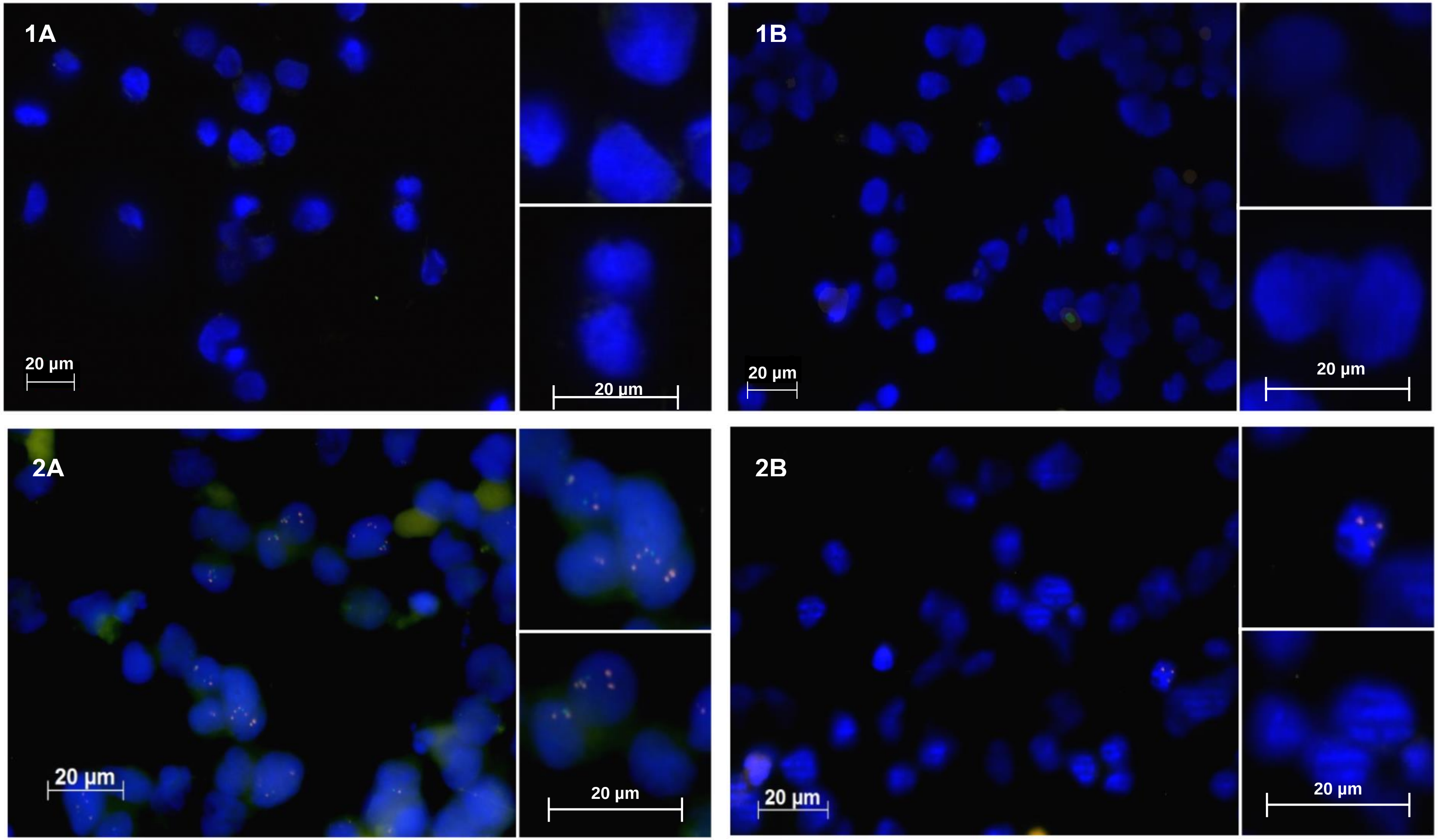

Supplementary Figure 3. *BRCA1* mRNA expression levels detected with RNAscope in a LCL containing *BRCA1* c. 671-2 A>G (panel 1a), and in a control LCL (panel 1b), and *BRCA2* mRNA expression levels in a LCL containing *BRCA2* c.7988 A>T (panel 2a), and in a control LCL (panel 2b), not treated with NMD inhibitors.

A. B.

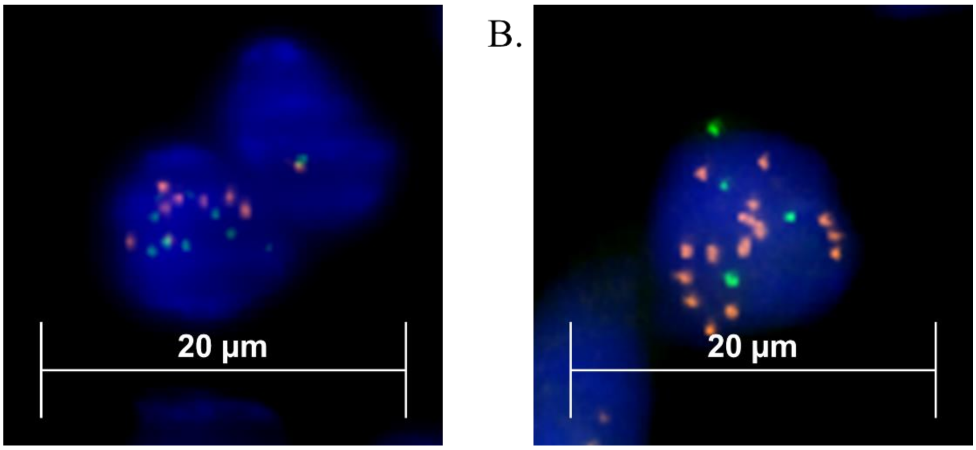

Supplementary Figure 4. Examplar highlighting how the differences in the number of each probe detected in a cell is indicative of the number of mRNA isoforms that carries the targeted deletion. A: cellular mRNA that does not carry the targeted deletion showing similar numbers of both green (C1) and red (C2) signals, indicating a background level of exon skipping. B: cellular mRNA that contains the deletion of interest, with a greater number of red signals (C2; total number of mRNA expressed) compared to green signals (C1; targeting the deletion), indicating a higher frequency of exon skipping than seen in wild type cells.
